# Air pollution and neurological diseases, current state highlights

**DOI:** 10.3389/fnins.2024.1351721

**Published:** 2024-03-06

**Authors:** Raymond Roy, Amedeo D’Angiulli

**Affiliations:** ^1^Department of Neuroscience, Carleton University, Ottawa, ON, Canada; ^2^Children’s Hospital of Eastern Ontario Research Institute, Ottawa, ON, Canada

**Keywords:** air pollution, neurological diseases, Alzheimer’s disease, Parkinson’s disease, autistic spectrum disorder, anxiety, depression, neuroinflammation

## Abstract

This paper delves into the increasingly recognized yet complex relationship between air pollution and Neurological Diseases. Although the detrimental effects of air pollution on respiratory and cardiovascular health are well-documented, its impact on neurological and cognitive disorders is an emerging area of concern. In this mini review, we explore the intricate mechanisms by which various air pollutants, such as particulate matter, nitrogen oxides, and polycyclic aromatic hydrocarbons, contribute to neurological pathologies. The focus lies on the role of oxidative stress and inflammation in exacerbating conditions like Alzheimer’s disease and Parkinson’s disease. By unraveling these connections, the paper sheds light on the broader implications of environmental factors on neurological health and underscores the urgent need for policy interventions to mitigate air pollution’s impact on the nervous system.

## 1 Introduction: neurological diseases and air pollution

According to World Health Organization (2021), neurological disorders encompass a wide range of ailments that impact the brain, spinal cord, and the body’s interconnected network of nerves. Alzheimer’s Disease, Parkinson’s Disease, Autistic Spectrum Disorder, Anxiety, Depression, Neuroinflammation are all included in this wide group (Gross et al., 2021; World Health Organization, 2021).

**Figure 1:**
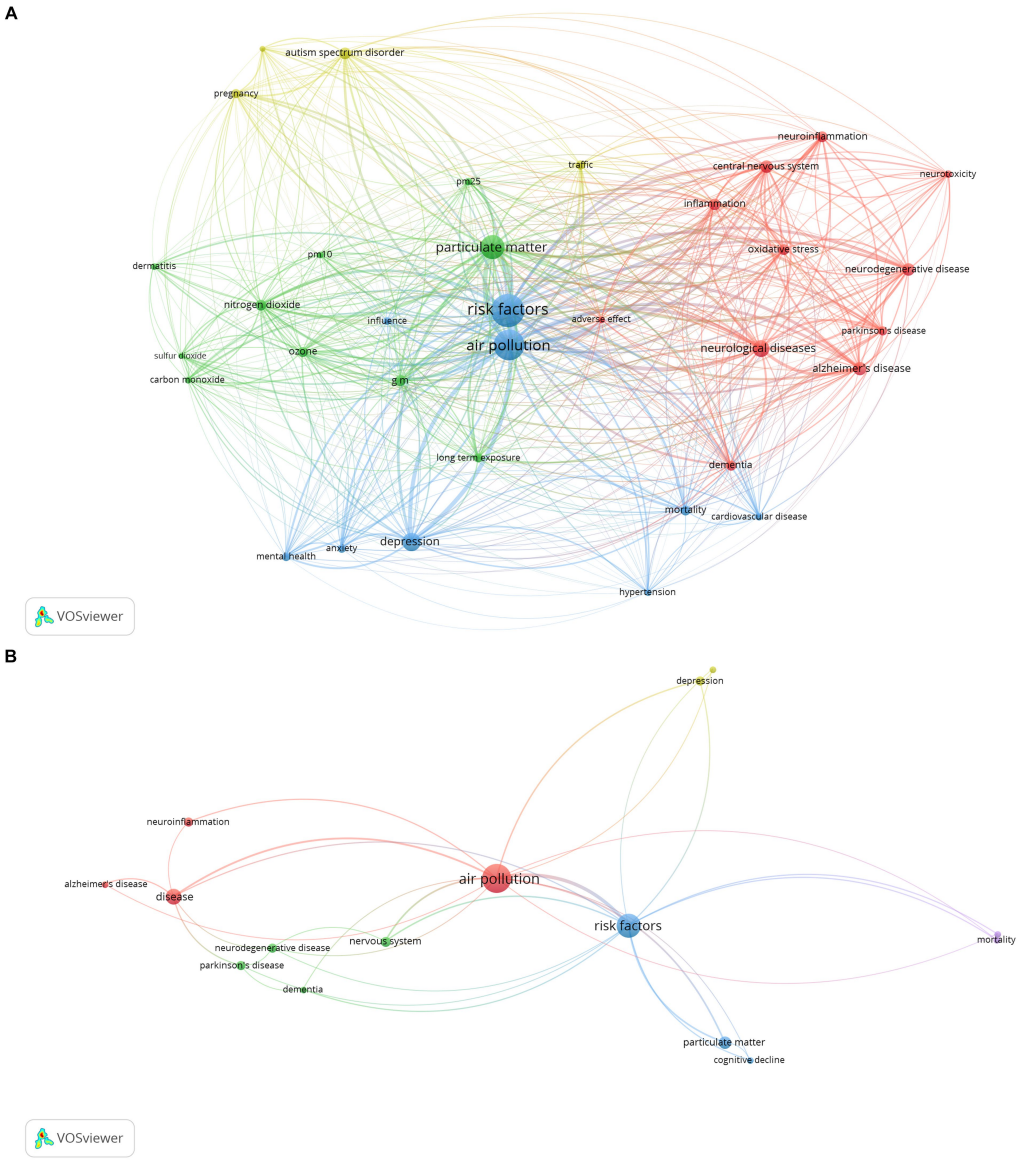
**(A)** A VosViewer network analysis of more than 1,600 PubMed papers focused on the relationship between neurological diseases and air pollution, as mentioned in the companion paper. With 34 items and a minimum keyword occurrence of 50, this visual map forms 4 distinct clusters with a total link strength of 19,343. This empirical data visually represents the current status of the field’s knowledge, highlighting the many connections and the prevalent emphasis on how air pollution affects neurological health. **(B)** A VosViewer network visualization illustrating the multifaceted linkages between air pollution and various neurological diseases. The network comprises 15 terms clustered into five groups with a total link strength of 77, reflecting the empirical evidence of air pollution’s role in neurological morbidity and mortality, Central to the network is ‘air pollution, a node demonstrably connected to increased risks and exacerbation of diseases like Alzheimer’s, Parkinson’s, Dementia, Anxiety, and Depression. This figure encapsulates the complex interactions and highlights the imperative for policy interventions targeting air pollution as a modifiable risk factor in the prevalence and progression of neurological diseases.

Figure 1A represents the current state of the entire domain of relationship between neurological diseases and air pollution according to an exhaustive bibliometric analysis including over 1,600 peer reviewed publications. This minireview highlights a subset of specific nodes, shown in Figure 1B, within the entire network of air pollution effects on the nervous system (for Figure 1 details, including the complete supplementary reference lists see Roy and D’Angiulli, 2024a,b).

### 1.2 Types of air pollutants

Air pollution is defined here by the effects of contaminants such as gases, chemicals, and particle matter. The various forms of air pollution, such as sulfur dioxide (SO_2_), lead (Pb), ozone (O_3_), nitrogen oxides (NOx), particulate matter (PM), and carbon monoxide (CO), are important because of their varying causes and effects on the environment and human health. These pollutants come from a mix of natural causes like volcanic activity and forest fires, as well as human activities like electricity generation, automobile emissions, and industrial processes (Timlin et al., 2018).

Incomplete combustion of fossil fuels in automobiles, factories, and home heating systems is the main source of carbon monoxide (CO). Because CO can attach to hemoglobin in the blood, it reduces the amount of oxygen that reaches the body’s tissues and organs Hanley and Patel (2019).

Lead (Pb) emissions have been greatly reduced in many areas due to regulations, particularly the removal of lead from gasoline. However, lead can still be found in industrial emissions and can accumulate in the environment, posing risks to human health, especially in children, by affecting neurological development (Dignam et al., 2019).

During combustion, particularly at high temperatures, the interaction of nitrogen and oxygen gases in the air produces nitrogen oxides (NOx), which include nitric oxide (NO) and nitrogen dioxide (NO_2_). These gases can worsen asthma, cause respiratory issues, and produce particulate matter and ozone at ground level (Bernabeo et al., 2019).

Sunlight reacts with pollutants like NOx and volatile organic compounds (VOCs) to produce ozone (O_3_) at ground level. Ozone shields humans from the sun’s UV rays while it is in the stratosphere, but at ground level, it is a dangerous pollutant that can lead to respiratory disorders and other health concerns (Soares and Silva, 2022).

Allergens, organic compounds, metals, dust or soil particles, and acids (such as nitrates and sulfates) are among the complex mixture of microscopic particles and liquid droplets that make up particulate matter (PM) in the air. Finer particles are more hazardous since they can enter the circulation and lungs deeply. PM can harm the heart as well as the lungs. Additionally, the size of these particles plays a crucial role in their impact on health, with PM2.5 (particles smaller than 2.5 μm) and PM10 (particles smaller than 10 micrometers) being of particular concern due to their ability to penetrate deeply into the respiratory tract and bloodstream (Schraufnagel, 2020).

Power stations and other industrial facilities produce sulfur dioxide (SO_2_) when they burn fossil fuels. It significantly affects health, exacerbating cardiovascular disorders and causing respiratory problems. Additionally, SO_2_ can combine with other elements in the environment to produce hazardous particles (Khalaf et al., 2022).

### 1.3 Mechanisms of neurological diseases in relation to air pollution

At the core of the connection between air pollution and Neurological Diseases lies the interplay between oxidative stress and inflammation (Brockmeyer and D’Angiulli, 2016). Several pollutants, including particulate matter (PM), nitrogen oxides (NOx), and polycyclic aromatic hydrocarbons (PAHs), are known to generate reactive oxygen species (ROS). This oxidative stress triggers an immune response, which, in turn, can harm cellular structures. The phenomenon of oxidative stress plays a pivotal role in the development of neurodegenerative disorders, including Alzheimer’s disease (AD) and Parkinson’s disease (PD) (Uttara et al., 2009).

For instance, exposure to PM2.5 has been strongly linked to increased markers of oxidative stress, microglial activation in the brain, and elevated pro-inflammatory cytokines (Allen et al., 2017). These findings suggest a significant contribution of PM2.5 to neurological pathology (Allen et al., 2017).

Another critical mechanism through which air pollution influences neurological disorders is the impairment of the blood–brain barrier (BBB). Prolonged exposure to air pollution can alter gene expression related to the BBB’s integrity and functionality, thereby increasing its permeability (Borroni et al., 2022).

Neurotoxic effects of air pollution go beyond oxidative stress and inflammation. Research indicates that PM can induce apoptosis in neuronal cells and hinder the differentiation of neural stem cells, which are essential for neuron development and brain function (Lee et al., 2023). Animal studies also suggest a link between air pollution exposure and brain damage, including neuron death and the formation of neurofibrillary tangles, a hallmark of AD. Furthermore, inhalation of PM particles has been associated with reduced neurogenesis in the hippocampus, a region crucial for learning and memory (Lee et al., 2023).

## 2 The exploration of air pollution’s influence on the nervous system

The exploration of air pollution’s influence on the nervous system reveals a complex interplay of mechanisms, notably oxidative stress, inflammation, impairment of the blood–brain barrier, and direct neurotoxic effects. These factors contribute significantly to the onset and progression of neurological disorders (de Prado Bert et al., 2018).

The evidence points to pollutants like particulate matter and polycyclic aromatic hydrocarbons as key contributors to neurodegenerative processes, affecting brain functions such as neuron development, memory, and learning (Lee et al., 2023). This understanding not only deepens our comprehension of the environmental determinants of neurological health but also underscores the urgent need for strategies to mitigate the impact of air pollution (Khreis et al., 2022).

### 2.1 Cognitive decline and neurodegeneration: a possible link to air pollution

In recent studies, air pollution has been linked to cognitive decline and neurodegenerative diseases, such as Parkinson’s disease (PD) and Alzheimer’s disease (AD) (Allen et al., 2017). This growing body of research underscores the urgent need to explore the impact of air pollution on mental health and public safety.

Particulate matter (PM) has been identified as a significant contributor to health issues (Lee et al., 2023). PM2.5, particles smaller than 2.5 μm, can penetrate deep into the lungs and bloodstream, posing serious health risks. Studies have linked PM exposure to cognitive deterioration and neurodegeneration due to inflammation, oxidative stress, and disruption of the blood–brain barrier.

In Mexico City, a study revealed that elderly adults exposed to high levels of PM showed significant cognitive decline, particularly those predisposed to Alzheimer’s disease (Grande et al., 2021). Similarly, a study in the United States found that PM2.5 exposure adversely affected cognitive function in older women, increasing the risk of dementia (Power et al., 2016). These findings are corroborated by research from India and China, highlighting the global relevance of PM exposure in cognitive health.

The mechanisms linking air pollution to cognitive decline are complex. Inflammation and oxidative stress caused by pollutants can damage neurons, while disruption of the blood–brain barrier facilitates the entry of harmful substances into the brain. Additionally, air pollution can lead to the accumulation of neurotoxic metals and beta-amyloid plaques, associated with Alzheimer’s disease (Kim et al., 2020).

Nitrogen dioxide (NO_2_), another prevalent air pollutant, has been associated with cognitive decline and neurodegeneration (Liu et al., 2016). NO_2_ can induce oxidative stress and inflammation in the brain, leading to neuronal damage. Studies have demonstrated that prolonged exposure to NO_2_ is linked to reduced cognitive abilities, particularly in the elderly.

Regarding Parkinson’s disease, studies have found a relationship between air pollution and the onset of PD (Lee et al., 2022). Exposure to PM2.5 and traffic-related pollution increases the risk of PD by inducing neuroinflammation and oxidative stress, leading to the degeneration of dopaminergic neurons.

### 2.2 Depression and anxiety

The growing concern about air pollution’s impact on mental health has brought to light its potential role in the development of depression and anxiety disorders. This connection highlights the complex interplay between environmental factors and neurological health (Borroni et al., 2022).

Depression, a widespread and debilitating psychiatric disorder characterized by persistent sadness and loss of interest, has been increasingly linked to air pollution. Studies suggest that components of air pollution such as nitrogen oxides, particulate matter (PM), and sulfur dioxide contribute to this condition. These pollutants can induce oxidative stress, leading to inflammation in the brain and subsequent structural changes that may precipitate depressive symptoms (Calderón-Garcidueñas et al., 2014).

In China, long-term exposure to PM2.5 was associated with higher levels of depression, while a German study linked traffic-related air pollution to an increased risk of depressive symptoms (Block and Calderón-Garcidueñas, 2009). Further supporting this, a meta-analysis found significant correlations between PM10, nitrogen dioxide, and depression (Borroni et al., 2022). This relationship may stem from the pollutants’ ability to increase reactive oxygen species, causing oxidative stress and inflammation in the brain, leading to neuron damage or disruption in neurotransmitter systems (Tran and Miyake, 2017). Additionally, prolonged exposure can impair neurogenesis and alter neuroplasticity, increasing susceptibility to depressive symptoms (Hunter and McEwen, 2013).

Anxiety disorders, characterized by excessive worry or fear that disrupts daily life, can also be exacerbated by air pollution. Pollutants may trigger the body’s stress response systems, including the hypothalamic–pituitary–adrenal (HPA) axis and the sympathetic nervous system (SNS), leading to heightened anxiety symptoms (Hunter and McEwen, 2013). Studies have shown that exposure to particulate matter and nitrogen dioxide increases anxiety-related symptoms (Hunter and McEwen, 2013).

The exact mechanisms by which air pollution leads to anxiety disorders are not fully understood, but several theories exist. Air pollution-induced inflammation in the brain may amplify anxiety symptoms (Block and Calderón-Garcidueñas, 2009). Additionally, pollutants can disrupt neurotransmitter balance in the brain, affecting serotonin and dopamine levels, which are crucial in regulating mood and anxiety (Tran and Miyake, 2017). Oxidative stress caused by air pollution is another factor linked to anxiety disorders (Tran and Miyake, 2017). Furthermore, exposure to air pollution may affect the gut-brain axis, influencing anxiety symptoms (Guxens et al., 2012).

### 2.3 Autism

The rising concern about air pollution has underscored its potential impact on public health, particularly regarding its link to the neurological and disorders like Autism Spectrum Disorder (ASD) (Becerra et al., 2013). ASD, a developmental disability marked by social interaction and communication difficulties, as well as repetitive behaviors, is increasingly viewed in the context of environmental health.

Studies suggest that air quality may play a significant role in the development of ASD, prompting a closer examination of this relationship. Research has shown a correlation between prenatal exposure to air pollutants and an increased risk of ASD. This link is further supported by research indicating a higher prevalence of prenatal developmental disorders, including schizophrenia, in polluted environments. A comprehensive meta-analysis corroborates these findings, highlighting the increased ASD risk associated with exposure during pregnancy (Power et al., 2016).

Anxiety is a prevalent issue among individuals with ASD, affecting about four out of every five people. This condition manifests through a combination of emotional and physical symptoms, such as elevated heart rate and muscle tension. The amygdala, a brain region essential for processing emotional information, is believed to play a crucial role in anxiety regulation in ASD. Recent research has explored the relationship between air pollution and anxiety in ASD individuals, finding that exposure to traffic-related air pollution was associated with heightened anxiety symptoms in children with ASD. Similarly, prenatal exposure to PM2.5, a particulate matter component, was linked to increased anxiety behaviors in children with ASD (Power et al., 2015).

In conclusion, the available research provides substantial evidence that air pollution may contribute to the development of ASD and the exacerbation of anxiety symptoms in individuals with this condition. This growing field of study is crucial for understanding environmental influences on developmental and mental health disorders, highlighting the importance of integrating environmental health perspectives in public health strategies (Guxens et al., 2012).

### 2.4 Parkinson’s disease

Parkinson’s Disease (PD) is closely linked to air pollution, as research has shown (Lee et al., 2022). PD, characterized by the degeneration of dopaminergic neurons in the substantia nigra of the brain, results in motor symptoms such as tremors, muscle stiffness, and balance issues. Recent studies suggest a potential connection between air pollution and an increased PD risk.

Air pollution, composed of various particulate matter and gaseous pollutants, has well-documented adverse effects on human health, including respiratory and cardiovascular diseases (Bont et al., 2022). Emerging evidence also points to its role in neurodegenerative conditions like PD.

Oxidative stress is a crucial mechanism through which air pollution may influence PD. It occurs when there’s an imbalance between the production of reactive oxygen species (ROS) and the body’s detoxification capacity (Liu et al., 2016). Airborne pollutants can exacerbate this imbalance, leading to neuronal damage, particularly in the dopaminergic pathways critical for PD. Chronic oxidative stress resulting from long-term exposure to air pollutants contributes to the progressive nature of PD.

Inflammation is another potential link between air pollution and PD. Inhaling polluted air can trigger brain inflammation, activating microglial cells, which can release pro-inflammatory cytokines (Palacios, 2017).

PD is a complex condition characterized by the accumulation of alpha-synuclein protein, forming Lewy bodies. Misfolded alpha-synuclein can be toxic to neurons and may be influenced by environmental factors like air pollution (Kinghorn et al., 2017). Additionally, mitochondrial dysfunction, a hallmark of PD, can lead to reduced ATP synthesis and increased oxidative stress, potentially exacerbated by environmental pollutants.

Anxiety, a common non-motor symptom in PD, can significantly impact patients’ quality of life. The causes of anxiety in PD are multifactorial and may involve neurotransmitter system changes influenced by external factors, such as air pollution (Power et al., 2015). Studies suggest that the general population’s anxiety due to air pollution exposure could also apply to individuals with PD, given their compromised neurological systems.

## 3 Structural changes in the brain and alterations in neurotransmitters and inflammation due to air pollution

The detrimental effects of air pollution on public health, especially the nervous system, have raised significant concerns. Recent studies indicate that prolonged exposure to polluted air can lead to structural changes in the brain, alterations in neurotransmitters, and increased inflammation, contributing to various neurological disorders, including anxiety disorders (Costa et al., 2020).

Air pollution has been associated with structural brain changes, such as reduced fractional anisotropy (FA) and increased mean diffusivity (MD), indicating a decline in white matter integrity, a sign of neurodegeneration (Lee et al., 2023).

Moreover, air pollution can lead to alterations in neurotransmitters. Exposure to polluted air has been linked to decreased dopamine and serotonin levels in the striatum, a region responsible for reward processing and motor control. Additionally, pollutants have been associated with reduced norepinephrine and dopamine levels, affecting executive functions in the prefrontal cortex (Krystal et al., 2002).

Fine particulate matter, such as PM2.5, has been found to be associated with a decline in gamma-aminobutyric acid (GABA) levels within specific brain regions responsible for emotional regulation and memory formation. This reduction in GABA, a neurotransmitter that promotes calmness and inhibits excessive neuronal activity, underscores the complex relationship between air pollution and its impact on the neural mechanisms underlying emotions and cognitive functions (Khreis et al., 2022).

Inflammation in the nervous system due to air pollution is a major concern. Exposure to PM2.5 has been linked to increased levels of inflammatory cytokines, such as interleukin-6 (IL-6) and tumor necrosis factor-alpha (TNF-α). Additionally, air pollution exposure leads to heightened microglial activation, indicative of neuroinflammation, in various brain regions (Armas and D’Angiulli, 2022; Zundel et al., 2022).

Research on the association between air pollution and anxiety is growing. Studies suggest that exposure to polluted air increases the risk of developing anxiety disorders, particularly in women (Power et al., 2015).

## 4 Discussion

Air pollution poses a significant ecological dilemma, affecting human health in various ways (Allen et al., 2014). Recent evidence suggests that it not only links to respiratory and cardiovascular diseases but also affects the nervous system, increasing the risk of neurological disorders like anxiety and depression (Zundel et al., 2022).

Anxiety, a physiological and psychological response to stress or danger, can manifest in symptoms like elevated heart rate, sweating, and breathing difficulties. Chronic anxiety can lead to social withdrawal and cognitive impairment. The origin of anxiety involves genetic, environmental, and social factors and includes various brain regions and neurotransmitter systems (Hunter and McEwen, 2013).

Air pollution may exacerbate anxiety through pathways like inflammation and oxidative stress. Inflammatory responses in the brain to air pollution can modify neurotransmitter systems and emotional regulation circuits. Oxidative stress, induced by reactive oxygen species generated by air pollution, can damage neurons and disrupt cellular function, contributing to anxiety and other neurological disorders (Zundel et al., 2022).

Compounds like curcumin, found in turmeric, have shown neuroprotective, anti-inflammatory, and antioxidant properties. Studies indicate that curcumin can mitigate neurological damage caused by air pollution, reducing oxidative stress and inflammation and improving cognitive function (Yi et al., 2020).

Resveratrol, present in grapes and other fruits, possesses antioxidant and anti-inflammatory properties beneficial for the nervous system. Research demonstrates that resveratrol can improve cognitive function by reducing oxidative stress and inflammation caused by air pollution (Cao et al., 2018).

Omega-3 fatty acids, known for their anti-inflammatory and neuroprotective capabilities, have been studied for their potential to counteract the effects of air pollution on the nervous system. Studies have shown that omega-3 supplementation can decrease oxidative stress and inflammation in the brain, enhancing cognitive function (Swanson et al., 2012).

Given the role of inflammation and oxidative stress, compounds like curcumin, resveratrol, and omega-3 fatty acids offer potential avenues for mitigating the harmful effects of air pollution on the nervous system (Khreis et al., 2022). Alongside reducing exposure to pollutants, adopting a healthy lifestyle, including regular exercise, a nutritious diet, and stress-reduction techniques are all ways to mitigate the effects we have reported. However, all these measures are not a substitute for actions aimed at reducing air pollution at its source, which remains the most effective way to protect community health.

## Author contributions

RR: Conceptualization, Resources, Writing – original draft, Writing – review & editing. AD’A: Conceptualization, Supervision, Writing – review & editing.
